# Ectopic Expression of Human *BBS4* Can Rescue Bardet-Biedl Syndrome Phenotypes in *Bbs4* Null Mice

**DOI:** 10.1371/journal.pone.0059101

**Published:** 2013-03-15

**Authors:** Xitiz Chamling, Seongjin Seo, Kevin Bugge, Charles Searby, Deng F. Guo, Arlene V. Drack, Kamal Rahmouni, Val C. Sheffield

**Affiliations:** 1 Department of Pediatrics, University of Iowa Interdisciplinary Program of Genetics, Iowa City, Iowa, United States of America; 2 Department of Ophthalmology and Visual Sciences, University of Iowa Carver College of Medicine, Iowa City, Iowa, United States of America; 3 Howard Hughes Medical Institute, Chevy Chase, Maryland, United States of America; 4 Department of Internal Medicine, University of Iowa Carver College of Medicine, Iowa City, Iowa, United States of America; 5 Department of Pharmacology, University of Iowa Carver College of Medicine, Iowa City, Iowa, United States of America; IGBMC/ICS, France

## Abstract

Bardet-Biedl syndrome (BBS) is a genetically heterogeneous autosomal recessive disorder characterized by obesity, retinal degeneration, polydactyly, hypogenitalism and renal defects. Recent findings have associated the etiology of the disease with cilia, and BBS proteins have been implicated in trafficking various ciliary cargo proteins. To date, 17 different genes have been reported for BBS among which *BBS1* is the most common cause of the disease followed by *BBS10*, and *BBS4*. A murine model of *Bbs4* is known to phenocopy most of the human BBS phenotypes, and it is being used as a BBS disease model. To better understand the in vivo localization, cellular function, and interaction of BBS4 with other proteins, we generated a transgenic *BBS4* mouse expressing the human *BBS4* gene under control of the beta actin promoter. The transgene is expressed in various tissues including brain, eye, testis, heart, kidney, and adipose tissue. These mice were further bred to express the transgene in *Bbs4* null mice, and their phenotype was characterized. Here we report that despite tissue specific variable expression of the transgene, human *BBS4* was able to complement the deficiency of *Bbs4* and rescue all the BBS phenotypes in the *Bbs4* null mice. These results provide an encouraging prospective for gene therapy for BBS related phenotypes and potentially for other ciliopathies.

## Background

Bardet-Biedl syndrome (BBS) is a pleiotropic, autosomal recessive disorder with the primary clinical features of obesity, retinopathy, polydactyly, learning disabilities, hypogenitalism, and renal defects [1], [2], [3]. BBS is rare in the general population: The prevalence in most of North America and Europe is 1 in 140,000 to 1 in 160,000 newborns [4]. However, components of the BBS phenotype are common, and debilitating including retinopathy, renal failure and obesity.

BBS is a genetically heterogeneous disease, and 17 different causative genes have been reported to date. Among these 17 genes, *BBS1* and *BBS10* have been reported to be the most common cause of the disease [5]. The data collected from our lab shows that *BBS4* is the third most common cause of this disorder. *BBS4* along with *BBS5* and *BBS8* are more common in patients of Middle Eastern and North African descent [6]. *BBS4* was the third BBS genes to be identified, after *BBS6* and *BBS2*, and its localization and interaction was analyzed soon after [7]. To better understand the function of BBS4, a Knockout murine model was generated and shown to phenocopy most of the human BBS phenotypes [8]. Indeed, *Bbs4−/−* mice have obesity, retinal degeneration, primary cilia dyskenisia, and lack spermatozoa flagella [8]. *Bbs4^−/−^* mice are also hypertensive, leptin resistant and have renal defects [9]. Due to these significant phenotypes, *Bbs4^−/−^* mice are commonly used as a BBS disease model.

Structurally, BBS4 is comprised almost entirely of tetratricopeptide repeats (TPR) that are predicted to fold into rod-shaped alpha solenoids [10]. Human *BBS4* shares 89% similarity with mouse and 81% with zebrafish. BBS4 has been shown to form a complex known as the BBSome with six other BBS proteins [1]. In addition, BBS4 has been shown to interact with centrosomal protein PCM1 and potentially with p150^glued^ [7]. Although the function of the BBSome complex has been studied, the role of BBS4 as a part of the PCM1 complex is not well understood. Moreover, due to the abundance of proteins in and around cilia where BBS4 localizes, it is likely that BBS4 interacts with other proteins that remains to be indentified. To better understand the in vivo localization and cellular function of BBS4, and to indentify novel interacters of BBS4, we generated a transgenic *BBS4* (*BBS4^tg^*) mouse expressing the human *BBS4* gene under control of the ß-actin promoter. Here we report that despite tissue specific variable expression of the transgene, human *BBS4* was able to complement the deficiency of *Bbs4* and rescue all BBS phenotypes when crossed onto the *Bbs4* null genetic background.

## Results

### Generation of Transgenic BBS4

We generated transgenic mice carrying human *BBS4* tagged with a lap tag (localization and purification tag) under the control of β-actin promoter (fig. S1A), which is known to drive consistently strong, and ubiquitous expression. A lap tag consists of GFP and S tag flanking a TEV cleavage site. We used a pHβApr expression vector to clone the N-terminally tagged *BBS4*. Expression of the construct was confirmed by transient transfection of the plasmid in 293T cells followed by western blotting as well as immunoflourescence (data not shown). Transgenic mice were generated using pronuclear injection [11] by the Transgenic Animal Facility at the University of Iowa. Several founder mice were obtained, and a transgenic line that most broadly expressed the highest level of LAP-BBS4 was selected for further study. We crossed mice carrying the human *BBS4* transgene (*BBS4^tg^*) with *Bbs4^+/−^* mice to generate (*BBS4^tg^/Bbs4^+/−^*) mice. These mice were crossed to generate *BBS4^tg^/Bbs4^−/−^* mice and other combinations of genotypes for the study (table 1). In the following sections *Bbs4^+/+^* are referred to as WT, *Bbs4^−/−^* mice are referred to as KO, and *(BBS4^tg^/Bbs4^−/−^)* are referred to as TG.

**Table 1:** A table showing combination of male and female of various genotypes crossed, their litter size and total number of pups produced.

Dam ID		Dam genotype	Sire ID	Sire genotype	# Litters	Total Pups	# Born Dead
*	*(1291-4)-53*	Bbs4^ wt^Bbs4^+/+^	*(B4T78-2)-33*	Bbs4^ wt^Bbs4^+/+^	7	37	**0**
	*(B4T78-4)-17*	Bbs4^ wt^ Bbs4^+/−^	*(B4T78-4)-14*	Bbs4^ tg^Bbs4^+/+^	6	54	0
	*(B4T78-5)-20*	Bbs4^ wt^ Bbs4^+/−^	*(B4T78-5)-17*	Bbs4^ tg^Bbs4^+/+^	3	21	0
	*(1294-16)-3*	Bbs4^ wt^ Bbs4^+/−^	*(B4T78-1)-23*	Bbs4^ tg^Bbs4^+/+^	4	21	0
	*(1294B4T-1)-1*	Bbs4T^ wt^ Bbs4^+/−^	*(1294B4T-1)-6*	Bbs4^ tg^Bbs4^+/−^	7	49	0
Ψ	*(1294B4T-2)-5*	Bbs4^tg^ Bbs4^−/−^	*(1294B4T-1)-7*	Bbs4^ tg^Bbs4^−/−^	6	47	0
Ψ	*(1294B4T-2)-19*	Bbs4^tg^ Bbs4^−/−^	*(1294B4T-2)-9*	Bbs4^ tg^Bbs4^−/−^	10	84	0
	*(1294B4T-2)-18*	Bbs4^tg^ Bbs4^+/−^	*(1294B4T-2)-12*	Bbs4^ tg^Bbs4^+/−^	4	41	0
	*(1294B4T-3)-14*	Bbs4^tg^ Bbs4^+/−^	*(1294B4T-5)-20*	Bbs4^ wt^Bbs4^+/−^	7	38	0
	*(1294B4T-6)-28*	Bbs4^tg^ Bbs4^+/−^	*(1294B4T-6)-22*	Bbs4^ tg^Bbs4^+/−^	3	21	0
Ψ	*(1294B4T-4)-51*	Bbs4^wt^ Bbs4^−/−^	*(1294B4T-4)-46B*	Bbs4^ tg^Bbs4^−/−^	3	15	0

Rows indicated by (Ψ) are the matings between male *BBS4^tg^/Bbs4^−/−^* and female *BBS4^tg^/Bbs4^−/−^* or *Bbs4^−/−^* animals; their litter sizes and the pups produced are also shown. No abnormality was found in litters produced or number of pups produced compared to the control matings (*). Genotyping was performed for *LAP-BBS4* (*BBS4^tg^*) as well as endogenous *Bbs4* as shown in (fig. S1B).

### Expression of the Transgene (LAP-BBS4)

**Figure 1:**
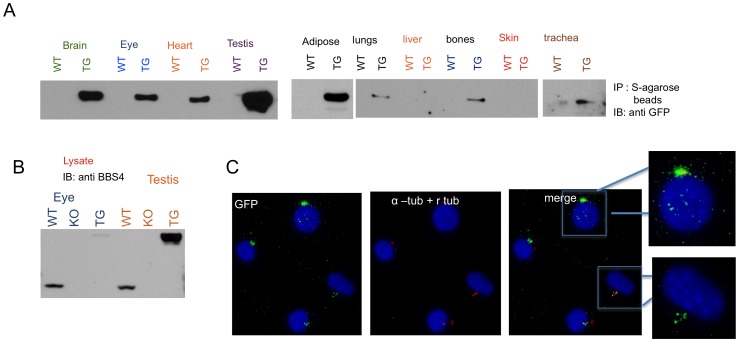
LAP-BBS4 is expressed in multiple different tissues. Due to multiple non-specific bands, comparing the expression of LAP-BBS4 to endogenous Bbs4 using protein lysates was not feasible except in eye and testis. **A)** For the other tissues, we used S-agarose beads to pull down the LAP-BBS4 from the lysates and immunoblotted with GFP antibody. The bands indicate the presence of LAP-BBS4, which is missing in the WT mice. **B)** Protein lysates from eye and testis of WT, *Bbs4^−/−^*, and *BBS4^tg^* animals were run on SDS gels and immunoblotted with BBS4 antibody. The upper band shows the amount of LAP-BBS4, and the lower band is the endogenous Bbs4. **C)** Localization of LAP-BBS4 was studied using immunoflourescence in MEF cells prepared using transgenic mice. Cilia were stained with acetylated α-tubulin and LAP-BBS4 was stained using GFP. As expected the centrosomal and ciliary localization of the LAP-BBS4 was observed.

Although a strong and ubiquitous promoter was used, expression of the LAP-BBS4 was limited to the few tissues, where endogenous Bbs4 is highly expressed. We analyzed the expression of the transgene via western blot. Comparing the expression of endogenous Bbs4 to the LAP-BBS4 using western blotting was not feasible since it was hard to get clean blot from a tissue lysate except in eye and testis (fig. 1B). When equal amount of lysate was loaded, lower expression of the transgene in the eye and higher expression in the testis, compared to the endogenous Bbs4, was observed (fig. 1B). For the other tissues, we used S-agarose beads to pull down and test which tissues express the LAP-BBS4 (fig. 1A). Highest expression of the transgene was found in testis, followed by brain, adipose, eye, heart, and very slight expression in trachea, lungs and bone; no expression in the liver and skin was observed.

We also performed rtqPCR to compare the amount of *LAP-BBS4* RNA expressed in transgenic mice compared to endogenous *Bbs4* expressed in WT mice (fig. S1C). Only tissues with robust observed based on western blotting were used for rtqPCR. Since immunohistochemisty (IHC) was not able to detect the transgene in the brain, different brain regions were also included in rtqPCR to better understand which brain region has the transgene expression. The level of *LAP-BBS4* RNA compared to endogenous *Bbs4* was higher in testis, and in regions within the brain including hypothalamus and cerebellum, but lower in other regions of the brain and also other tissues including eye, kidney, and adipose tissue (fig. S1C). Due to low expression of BBS proteins and lack of a good functioning antibody, it is difficult to stain tissues with BBS4. Therefore, to confirm that the transgenic protein product (LAP-BBS4) localizes similarly to the endogenous Bbs4 in our transgenic animals, we performed immunoflourescence, on MEF cells prepared from the transgenic animal; as we expected, the localization of LAP-BBS4 is similar to endogenous BBS4 such that it localizes to the centrosome and cilia (fig. 1C).

### Expression in Testis Rescues Male Infertility

**Figure 2:**
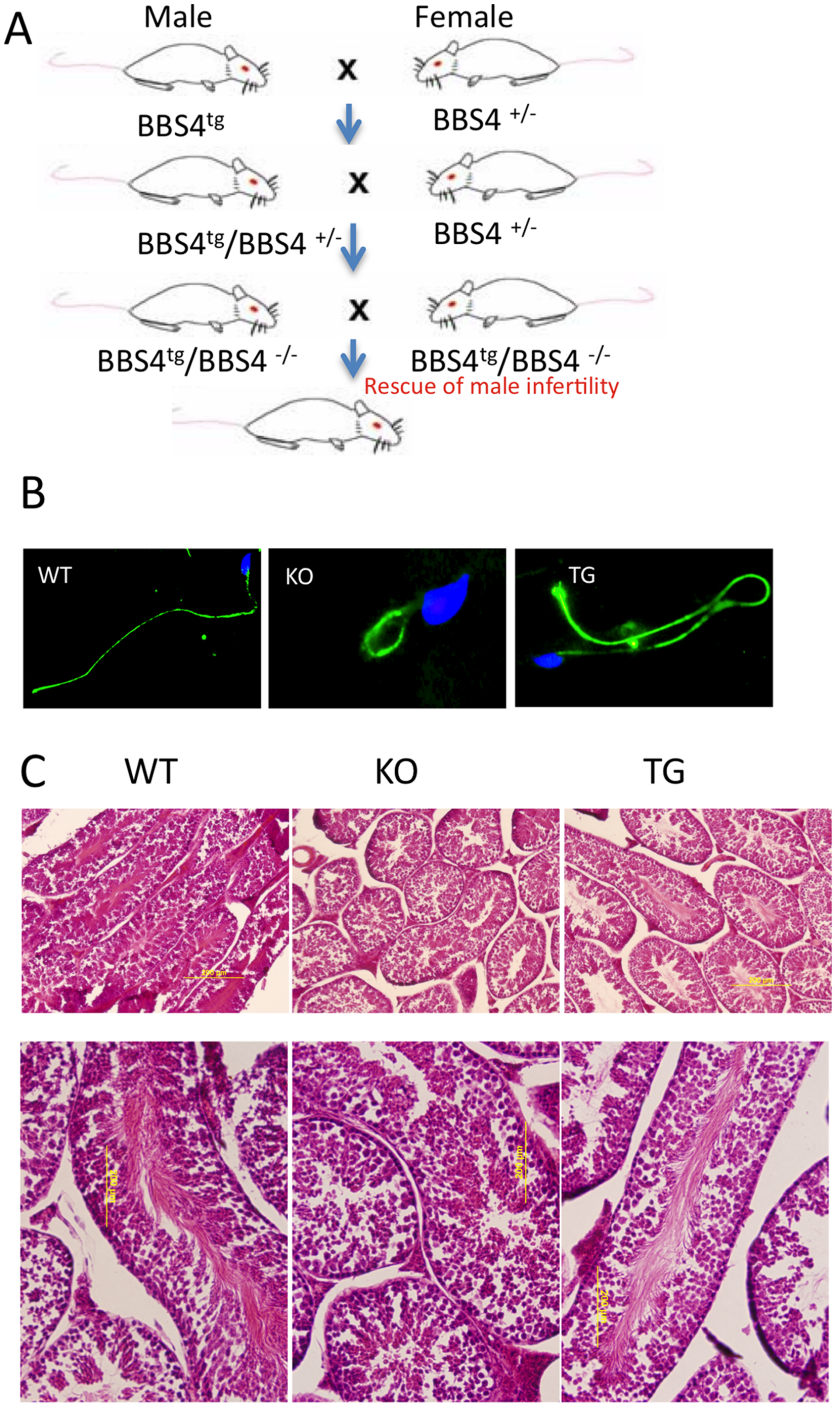
Infertility in *Bbs4^−/−^* mice is rescued by LAP-BBS4. A) We started with male *Bbs4^tg^* mice crossed with female *Bbs4^−/−^* mice. In the F3 generation, we selected male transgenic animals lacking endogenous *Bbs4* (*BBS4^tg^/Bbs4^−/−^*) and crossed them with female *Bbs4^−/−^.* Male *BBS4^tg^/Bbs4^−/−^* mice were able to produce pups indicating rescue of the infertility. **B)** Sperm flagella are normal in *BBS4^tg^* mice. Sperm heads are stained with DAPI, while the sperm tails are stained with acetylated a tubulin. *Bbs4^−/−^* male mice produce spermatozoa that have either no flagella or in a few cases short, truncated flagella. Sperm tails in *BBS4^tg^* animals are normal and full length. **C)** H&E staining show the testis histology demonstrating diminished sperm production in *Bbs4^−/−^* testis compared to WT and the *BBS4^tg^* animals.

Loss of sperm flagellum leading to male infertility is one of the most evident phenotypes in the *Bbs4^−/−^* mice [8]. To test if the transgene can act to rescue the deletion of the endogenous *Bbs4* and reverse the male infertility, we crossed male transgenic mice, *BBS4^tg^* to female *Bbs4^+/−^* mice. Resulting transgenic heterozygous males (*BBS4^tg^/Bbs4^+/−^*) were further crossed with heterozygous females *(Bbs4^+/−^)*. We selected the *Bbs4^−/−^* animals expressing the transgene *(BBS4^tg^/Bbs4^−/−^)* and crossed them again. The *BBS4^tg^/Bbs4^−/−^* male and female were able to breed normally and produce similar litter size and number of pups as the control mating (Fig. 2B, Table 1); the pups produced were either *BBS4^tg^/Bbs4^−/−^* or *Bbs4^wt^/Bbs4^−/−^.* Female *Bbs4^−/−^* mice are fertile, and the reason of infertility in the *Bbs4^−/−^* male mice is lack of sperm flagella, which are completely missing or severely decrease in size in the *Bbs4^−/−^* mice (Fig. 2C–D). We also analyzed the structure of the sperm flagellum, and histology of testis in these *BBS4^tg^/Bbs4^−/−^* males using immunoflouresence and H&E staining respectively, and we could see elongated, healthy, and normal sperm flagella, similar to the WT (fig. 2C–D).

### Low Expression of the BBS4 Transgene Rescues Retinopathy

**Figure 3:**
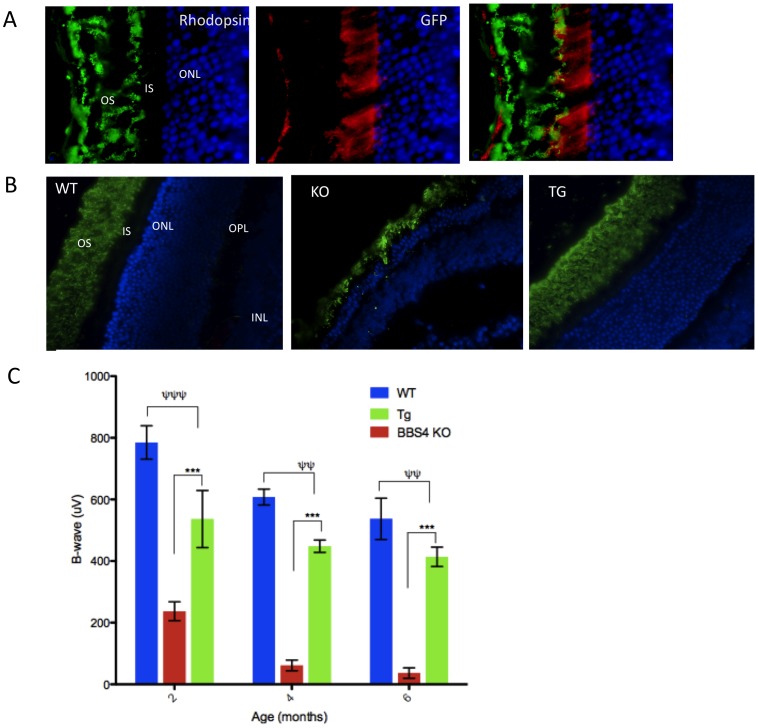
*LAP-BBS4* can significantly improve retinal degeneration in *Bbs4^−/−^* animals. **A)** LAP BBS4 is expressed in the inner segment of a retina (red) as observed using antibody against GFP. Green is outer segment rhodopsin staining and blue is nuclear staining with DAPI; slight LAP-BBS4 expression in the RPE layer is also seen. **B)** The histology of retina observed using rhodopsin (green) and DAPI (blue) staining in 2 months old mice show that *Bbs4^tg^* mice have normal retinas. **C)** ERG measured at 4 different time points in WT, *Bbs4^tg^*, and *Bbs4^−/−^* mice. Data shown are mean +/− SEM with n = 4 for each genotype. B-wave value shows that the ERG remains significantly different in the *BBS4^tg^* mice when compared to the WT (Ψ Ψ Ψ P<0.001, Ψ Ψ P<0.01), however, it is drastically and significantly improved in the *Bbs4^tg^* mice compared to the *Bbs4^−/−^* mice (***P<0.001). The difference between *Bbs4^tg^* and *Bbs4^−/−^* mice becomes more significant as the mice get older.

In the *Bbs4^−/−^* mouse model, the photoreceptors degenerate progressively with a loss of nuclei and shortening of the inner and outer segments leading to complete loss of photoreceptor cells overtime [12] (fig. 3B). Our transgenic mice have low transgenic expression in the eye (fig. 1, S1C). Within retina, expression of transgene was observed mostly in the inner segment of an eye with very low expression in the RPE layer (Fig. 3A). The qPCR data show that expression of the transgene is approximately 30 fold lower compared to endogenous *Bbs4* expression (fig. S1C). We performed electroretinography (ERG) on 2,4 and 6 months old mice to test whether the observed amount of transgene expression was able to rescue the blindness in *BBS4^tg^* mice. In WT eye, the average b-wave amplitude is approximately 800 uV at 2 months of age, whereas b-wave amplitude is drastically reduced to 200 uV in *Bbs4^−/−^* mice (fig. 3C). In *Bbs4^tg^* mice compared to the *Bbs4^−/−^* mice, the b- wave is significantly increased to approximately 550 uV. Although the ERG b-wave in *Bbs4^tg^* mice remained significantly different compared to the WT mice, the difference becomes less with age, which indicates prevention of retinal degeneration by the transgene. By the end of 6 months, the b-wave amplitude difference between the *Bbs4^−/−^* and *BBS4^tg^* mice was almost 5 fold (fig. 3C). We also performed histology on mouse eyes of different ages. The histology data from 2 months old *BBS4^tg^* mice (fig. 3B) show that the outer segment and ONL were preserved.

### Rescue of Obesity

**Figure 4:**
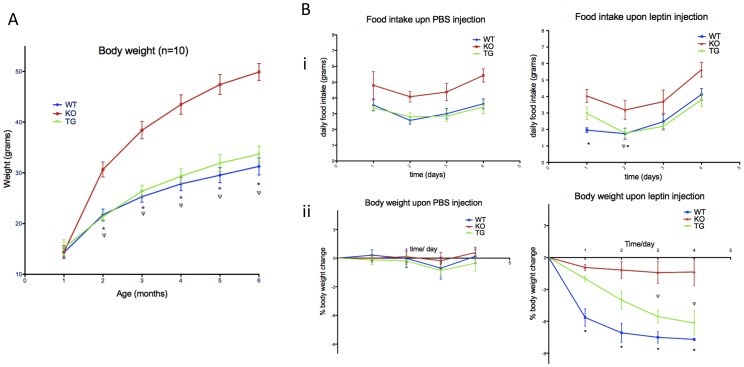
Obesity and leptin resistance in *Bbs4^−/−^* mice are rescued by *LAP-BBS4*. A) 10 mice per group **(**5 male and 5 female) mice were weighed every month to 6 months of age. *Bbs4^−/−^* mice (red line) become significantly heavier than WT mice (*P<0.01) and the *Bbs4^tg^* mice (Ψ P<0.01) beginning at 2 months of age. *Bbs4^tg^* mice (blue line) are not significantly heavier than WT mice (green line) at any age. Data are presented as mean +/− SEM, n = 10 per genotype. **B)** Weight change and food intake was measured in the WT (blue), *BBS4^tg^* (green), and *Bbs4^−/−^*(red) mice upon vehicle (PBS) or leptin injection**. i)** When compared to vehicle injection, food intake of WT mice was significantly reduced on day 1 and 2 after beginning leptin injection (*P<0.05), and the food intake of *BBS4^tg^* mice was decreased on day 2 after beginning leptin injection (Ψ P<0.05). T-test was used to calculate significance **ii)** Leptin injection had no significant effect on body weight or food intake in *Bbs4^−/−^* mice. When compared to the *Bbs4^−/−^*mice, WT mice lost significant amount of weight after day 1 of leptin injection (*P<0.01) and they progressively lost up to 7% of total body weight by the end of day 5. *Bbs4^tg^* mice when compared to *Bbs4^−/−^*mice, lost significant weight after day 3 of injection (Ψ P<0.01) and progressively lost up to 6% of body weight by the end of day 5. Four mice (2 males and 2 females) of each genotype were used for the study.

Obesity is one of the cardinal features of BBS [5]. Mouse models of BBS including *Bbs4^−/−^* mice become obese [8]. Since the *BBS4* transgene is expressed in the brain as well as in adipose tissues (fig. 1A), we expected obesity to be rescued in *Bbs4^−/−^* mice carrying the transgene. To test this hypothesis we weighed *Bbs4^−/−^*, WT, and *BBS4^tg^* mice every month to six months of age (fig. 4A, S2). *Bbs4^−/−^* mice were significantly heavier than WT and *BBS4^tg^* mice (P<0.01) by two months of age (fig. 4A, S2A). The weight difference increased with time. At six months of age the *Bbs4^−/−^* mice were more than 20(*+/−*3) grams heavier than WT mice. The weight gain is prevented in *BBS4^tg^* mice with no significant difference between these and WT mice (fig. 4A, S2A). Interestingly, we observed a difference in weight and onset of obesity in *Bbs4^−/−^* mice is also dependent on genetic background. Our *Bbs4^−/−^* mice on the 129/SvJ background become obese earlier and to a greater extent than *Bbs4^−/−^* mice on the C57 and 129 mixed background reported by Mykyten et al. [8].

### Leptin Resistance and Renal Sympathetic Nerve Activity (SNA)

We previously compared the effect of exogenous leptin in BBS *Bbs4^−/−^* and WT mice by administrating leptin and tracking weight loss as well as food intake [9]. Compared to WT mice, *Bbs4^−/−^* mice were resistant to leptin with no significant change in body weight or food intake with leptin administration. Since there is expression of the transgene in the brain and hypothalamus, we decided to test whether the transgene can rescue the leptin resistance phenotype seen in *Bbs4^−/−^* mice. We injected 1 ug of leptin or vehicle (PBS) per gram of body weight twice a day, and monitored the weight change as well as food intake (Fig. 4B, S2B). There was no significant weight loss upon vehicle injection in any of the mice (fig. 4b ii). Upon leptin injection, there was less than 1% body weight loss in the *Bbs4^−/−^* mice. Compared to the *Bbs4^−/−^* mice, WT mice lost significant weight (p<0.01) starting the day following first leptin injection. They progressively lost weight each day, and 7% of body weight was lost by the end of day 5. Although the change in body weight was not as drastic after the first day of injection, progressive loss was apparent in the transgenic mice as well. Compared to the *Bbs4^−/−^* mice, *BBS4^tg^* mice lost significant weight (P<0.01) by the third day after leptin injection was started. By the end of day 5, 6% (*^+/−^*1%) of the body weight was lost in *BBS4^tg^* mice (fig. 4B ii), which indicates that these mice respond to leptin, similar to WT mice. We also measured the food intake in mice upon vehicle or leptin injection (fig. 4B i). On average, WT and *BBS4^tg^* mice eat 3.5 grams/day, while *Bbs4^−/−^* mice eat 5 grams/day (fig. 4C). Upon leptin injection, there was no significant change in the food intake of *Bbs4^−/−^* mice. However, the food intake was reduced in WT after the first day of injection, and food intake slowly progressed back to normal (3.5 grams/day) by the end of day 5. Although the change in *Bbs4^tg^* mice was not significant on the first day, the food intake subsequently followed the same trend as in WT mice (fig. 4B i). When compared to food intake upon vehicle injection, food intake of WT mice significantly (P<0.05) decreased beginning on day 1 of leptin injection. In *BBS4^tg^* mice a significant reduction in food intake (P<0.05) was observed beginning on day 2 after leptin injection was started. In general, food intake and the response to leptin in *BBS4^tg^* mice were significantly different than the food intake and response to leptin of *Bbs4^−/−^* mice.

Previously, we observed that renal sympathetic nerve activity (SNA) was significantly greater in *Bbs4^−/−^* animals compared to the WT [9]. Average renal SNA in a WT mouse is 65+7 spikes/sec. Consistent with our previous finding, in *Bbs4^−/−^* mice, renal SNA is increase (79±19 spikes/sec). The renal SNA in the *BBS4^tg^* mice is 66±9 spikes/sec, which is not significantly different from WT mice (fig. S3A).

### Rescue of Hydrocephalus and Motile Cilia

**Figure 5:**
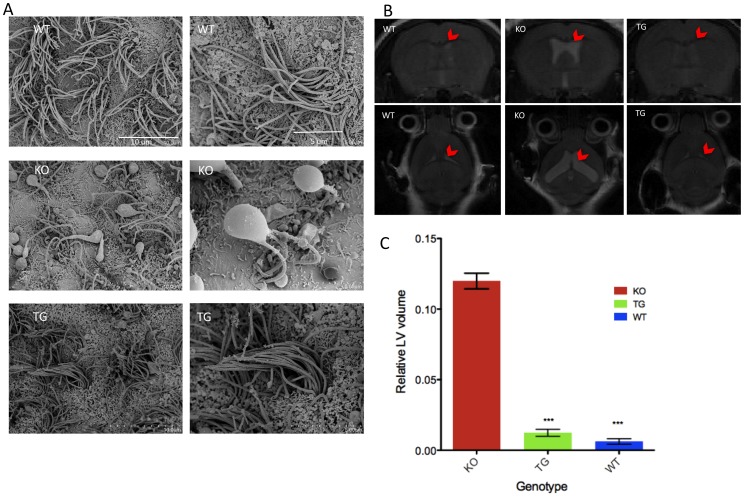
LAPBBS4 can rescue motile cilia defects and hydrocephalus. A) SEM image of cilia from the lateral ventricles of the brain in 6 months old WT, *Bbs4^−/−^*and *BBS4^tg^* mice. Motile cilia appear normal and healthy in *BBS4^tg^* mice. Right panel shows the motile cilia in higher magnification. **B)** *Bbs4^−/−^* mice have hydrocephalus (enlarged ventricles, arrowheads). The top panel shows an MRI of 3-month-old mouse brains in coronal section, and the bottom panel shows the horizontal sections of the same mice. The ventricles are slightly visible in the WT mice and in the *Bbs4^tg^* mice. **C)** Relative volume of lateral ventricle is significantly low (***P<0.001) in WT and *BBS4^tg^* mice compared to the *Bbs4^−/−^*mice (data shown as mean +/− SEM, n = 3 per genotype).

Hydrocephalus is observed in all BBS murine models, including *Bbs4^−/−^* mice. There is evidence linking ventricular motile cilia abnormalities to congenital hydrocephalus [13], [14]. Along with hydrocephalus, our BBS murine models have motile cilia defects in the ependymal cells lining the cerebral ventricles, as well as in airway epithelia [15], [16]. We performed scanning electron microscopy (SEM) to examine the cilia in the lateral ventricles of WT, *Bbs4^−/−^*, and *BBS4^tg^* mice (fig. 5A, S3B). *Bbs4^−/−^* mice have very few cilia and most of them are grossly deformed with large bulges at the tip when compared to WT mice. *BBS4^tg^* mice have normal numbers of cilia and the cilia are morphologically normal (fig. 5A, S3). The expression of the transgene in the brain is also capable of rescuing the hydrocephalus in *BBS4^tg^* mice. Coronal and horizontal MRI sections were taken in 3 months old WT, *BBS4^tg^* and *Bbs4^−/−^* mice (fig. 5C, S3B). The MRI images show enlarged lateral ventricles (LV) in the *Bbs4^−/−^* brain, but not in WT and *BBS4^tg^* brains (fig. 5B, S3C). Relative LV volume of WT and *BBS4^tg^* brain is significantly lower compared to *Bbs4^−/−^* (P<0.001) (fig. 5C).

## Discussion

This study was designed to better understand the localization, function, and interactions of BBS4, and to determine whether ectopic expression can functionally complement endogenous Bbs4. In our study, we used human *BBS4* with a LAP tag at the N-terminus of BBS4 and ectopically expressed the protein in mice lacking endogenous *Bbs4*. Previously; we generated similar transgenic mice with the *BBS4* gene controlled by the CMV promoter [17]. While we saw significant improvement in body weight and fertility, the transgene did not appear to be ubiquitously expressed [17]. In the mouse generated in this study, the transgene appeared to be more broadly expressed, although the level of expression varied among tissues. Despite an apparent low level of expression in some tissues, the human *BBS4* transgene was able to rescue almost all of the BBS phenotypes observed in *Bbs4* null mice.

Human and mouse BBS4 share 89% similarity, and both, almost entirely, consist of TRP repeats flanked by short N-and C-terminal regions [7], [18]. Each of the proteins contains 8 TPR repeats spanning from 67 amino acids to 371 amino acids. Therefore, we expected the human homologue of BBS4 to replicate the function of mouse BBS4. In fact, BBS4 interacting proteins including PCM1 and BBSome components are the same in human as well as mouse. BBS null mice demonstrate most of the human BBS phenotypes. However, there are some differences in the protein sequence including a 14 aa low complexity region at the N-terminus of the mouse protein that is absent in the human BBS4 protein (SMART database). Such differences could lead to differences in interacting partners and could account for some variation in phenotypes between mice and human. For example, polydactyly is a major feature of BBS in human patients, but it is not found in *Bbs* null mice. Furthermore, hydrocephalus is not highly penetrant in human patients, but is frequently observed in *Bbs* null mice. Of note, all observed mouse BBS phenotypes, including hydrocephalus were rescued in Bbs4 null mice by ectopic expression of human BBS4.

An important function of BBS proteins is ciliary proteins trafficking. In sperm, BBS proteins localize to the annulus and are thought to traffic essential proteins to form elongated sperm tails. In the retina, the BBS proteins localize to the ciliary body and assist in protein trafficking for maintenance of the outer segment of photoreceptors. We observed retinal degeneration, as well as either completely missing or only partially formed sperm flagella in *Bbs4^−/−^* mice. In addition, recent findings have implicated leptin resistance and defective leptin receptor b trafficking in the hypothalamus to obesity in *Bbs4^−/−^* mice (2, 9). The role of BBS proteins and the BBSome in protein trafficking has been widely studied in primary cilia using cell culture, but in-vivo confirmation is lacking. Using our transgenic mice we have now shown that ectopic expression of BBS protein can effectively restore sperm flagella formation, rescue obesity, and successfully restore rhodopsin trafficking in photoreceptor cells preventing retinal degeneration. By using this in-vivo model we have further confirmed the role of BBS proteins in ciliary trafficking.

The role of BBS proteins in motile cilia has not been as widely explored compared to the role in primary cilia. Shah et al [16] noted few deformed motile cilia in the airway epithelial cells in *Bbs* null mice. Deformed motile cilia at the ependymal lining of the ventricles are also seen in *Bbs3^−/−^* mice [15]), which could be the cause of hydrocephalus in these mice. *Bbs4* null mice also have these phenotypes. The ectopic expression of *BBS4* was able to correct the motile cilia defect in *Bbs4* null mice. The results indicate an important role of BBS proteins in protein trafficking in motile, as well as primary cilia.

In *Bbs4^tg^* mice, expression of LAP-BBS4 was highly variable among tissue types. High expression of LAP-BBS4 compared to endogenous mouse Bbs4 was seen in testis, while low expression was observed in the eye (fig. 1A, S1B). Within the brain, the cortex, hippocampus, and amygdala had lower expression levels compared to the hypothalamus and cerebellum. Expression of the transgene in the hypothalamus appeared higher than endogenous *Bbs4* expression (fig. S1B). Despite the variable and often weak expression of the transgene, there was rescue of BBS phenotypes, which suggests that the amount of BBS4 expression is less important than its presence. Collectively, our results suggest that the rescue of BBS phenotypes is not dose dependent, and almost all the known BBS phenotypes can be corrected by supplementing the gene.

Simons et al [19] reported that sub retinal injection of AAV with *BBS4* with coverage factor of as low as 5% was able to rescue the mislocalization of rhodopsin in *Bbs4* null mice and preserve the rod outer segment. In agreement with their data, we were able to show that a greater than 20-fold lower level of expression of transgene compared to endogenous Bbs4 expression was adequate to improve the ERG, rhodopsin localization and overall histology of the retina. Similarly, obesity in BBS has been associated with hyperleptinemia, which occurs due to leptin receptor mislocalization in hypothalamic neurons in the absence of BBS4 [9]. Our data shows that ectopic expression of human *BBS4* was able to reduce hyperleptinemia and rescue obesity in *Bbs4* deficient mice. The expression of the transgene in the brain was able to preserve motile cilia in the ventricles of the brain, and rescue hydrocephalus. Our results support the concept that syndromic obesity, retinopathy, hydrocephalus and other ciliary phenotypes are candidates for gene therapy.

## Methods

Ethics statement: The University Animal Care and Use Committee at the University of Iowa approved all animal work in this study (animal protocol number: 1003062). Every person involved in handing mice was properly trained to the standard proposed by the committee.

### Generation of Transgenic Mice

We generated transgenic mice carrying human *BBS4* tagged with the lap tag under the control of the β-actin promoter. We used a pHβApr expression vector [20], [21] to clone the N-terminally tagged *BBS4*. Transgenic mouse lines were generated using pronuclear injection [11] by the transgenic animal facility in University of Iowa. B6SJL (C57BL/6J X SJL/J: Jackson Laboratory) embryos were used for the pronuclear injection. Transgenic animals were maintained on 129/SvJ background. To generate *BBS4* ^tg^ */Bbs4* ^−/−^ and *Bbs4* ^−/−^ mice used in this study, *BBS4^tg^/Bbs4* ^+/−^ lines were crossed with *Bbs4* ^+/−^ animals on the 129/SvJ background. Genotypes for the *lap-BBS4* transgene were determined using the following primers: GTCCTGCTGGAGTTCGTGAC and GGCGAAATATCAATGCTTGG). For *Bbs4* ^−/−^ genotyping, the following primers were used: GCTACCCGTGATATTGCTGAA and TTGGGTGCTCTATTCTGCTG.

### Antibodies and Reagents

BBS4 antibody was kindly provided by Dr Maxence Nachury (Stanford University). Anti-acetylated tubulin (6-11B-1) was purchased from Sigma (St. Louis, MO, USA), GFP antibody (A11120), Alexa 488-conjugated and Alexa 568-conjugated secondary antibodies were purchased from Invitrogen (Carlsbad, CA, USA). S-agarose beads for immunoprecipitation were purchased from (novagen).

### Quantitative Real-time PCR, MEF Cells and Immunoflourescence Microscopy

For qPCR, RNA from different tissues was extracted using IBI RNA extraction kit (cat# IBI47302) following their protocol. cDNA was prepared from 2 ug of total RNA using SperScript III reverse transcriptase (Invitrogen). qPCR was performed using SYBR Green PCR Master Mix (Applied Biosystems # 4309155) in the Mx3000p unit (Stratagene). GAPDH was used to normalize the mRNA level and ΔΔCt [20], [22] method was used to calculate the expression of LAP-BBS4 compared to endogenous Bbs4. MEF cells from the transgenic mice was prepared from embryonic day 12.5 embryos. Pregnant mice were sacrificed by cervical dislocation following IACUC guidelines. Uterine horns of sacrificed mice were dissected out, and cultured in Dulbeco’s modified Eagle medium with 10% serum following the protocol by Jozefczuk et al. [23]. Immunoflourescence was performed as previously described (*22).*

### Tissue Sectioning, Staining, and SEM

For tissue sectioning, animals were perfused with 4% PFA in PBS (2.5 ml/min, 50 ml) following a standard protocol. Prior to perfusion, animals were fully anesthetisized by injecting Ketamine (0.1 ml/20 g BW). Eyes were post-dissection fixed with fixative for 30 more minutes and frozen in OCT. Sectioning was performed on the frozen section using CryoStar NX70 (thermo Scientific). Mouse sperms were extracted and stained as describe [17]. For brain, the tissue was freshly harvested without perfusion and snap frozen in liquid nitrogen. Frozen tissues were used for sectioning with the CryoStar. For SEM, animals were perfused as described above. Brain was harvested, and dissected to remove the hippocampus and expose the ventricle. The brains were then fixed with 2.5% glutaraldehyde for 24–48 hrs. Standard SEM protocol was followed to prepare the sample and a Hitachi S-4800 electron microscope was used for imaging.

### Obesity, Leptin Resistance, Food Intake and Body Weight Determination

Feeding and body weight response to leptin was compared between WT, *Bbs4^−/−^*, and *BBS4^tg^* animals as mentioned previously [9]. Mice were housed separately a week before the injection, and mock injections were performed as training for a week to eliminate stress and difference in environment as a cause of weight loss and differential food intake. One micro liter (1 mg/ml) of leptin per gram of body weight was given to each mouse via i.p injection twice a day for 4 days. Weight and food intake was measured every morning. For the obesity study, 10 mice (5 male and 5 female) from each genotype (WT, *Bbs4* ^−/−^ and *BBS4^tg^*) were weighed each month for up to 6 months. Littermates from 3 different parents were used for the study.

### ERG, and Renal SNA

For ERG measurement, mice were dark-adapted overnight prior to anesthetizing by ip injection with ketamine (87.5 mg/kg) and xylazine (2.5 mg/kg). Gold ring electrodes referenced to a needle electrode used to record ERG from corneal surface of each eye simultaneously following the description in [24]. To measure renal SNA, mice were anesthetized as mentioned above and a nerve fascicle to the left kidney was isolated and a bipolar platinum-iridium electrode was suspended as described in [9]. Baseline renal SNA was recorded for 10 min and an average of two separate measurements was used as the baseline value for each animal.

Statistics: Results are expressed as mean +/−SEM. Data was analyzed using 1-way ANOVA and TUKEY unless specified otherwise. P<0.05 is considered significant.

## Supporting Information

Figure S1 **Genotypes and relative expression of transgene varies among tissues. A)** Expression cassette of *LAP-BBS4*, where a LAP-tag is at the N-terminus of the *BBS4* gene. *LAP-BBS4* was cloned in phßAPr expression vector. **B)** Example of genotyping performed on the animals. Each animal was tested for the presence of the transgene as well as the endogenous *Bbs4* gene. Left side represents genotype for endogenous *Bbs4*, and the right side is the genotype for the transgene. **C)** Graph showing relative expression of the transgene (*LAP-BBS4*) compared to the endogenous *Bbs4*. The Y-axis is the relative fold change (*LAP-BBS4/msBbs4*), and the X-axis shows tissues that were used. Horizontal dotted line at 1 in Y-axis represents the point where equal expression of the transgene and endogenous *Bbs4* are observed. Above the line represents higher expression of *LAP-BBS4* compared to endogenous *Bbs4*, and below that reference line shows higher expression of endogenous *Bbs4* than transgene. For example, retina has relatively higher expression of endogenous *Bbs4* than *LAP-BBS4*, and higher amount of *LAP*-*BBS4* than the endogenous *Bbs4* is expressed in testis.
(TIF)

Figure S2 **Obesity and leptin resistance in** *Bbs4^tg^* **mice compared to** *Bbs4^−/−^* **mice**. **A)** 5 male and 5 female mice were weighed every month for 6 months. *Bbs4^−/−^* mice (red line) are significantly heavier than WT (*P<0.01)(green) and *BBS4^tg^* mice (Ψ P<0.01) beginning at 2 months of age. There is no significant difference in weight between *Bbs4^tg^* and WT mice in either male or female. **B)** Food intake and body weight were monitored in 4 mice (2 male 2 female each, 9–12 weeks old) of all three genotypes. Mice were kept in separate cages for a week followed by a training session of mock injection to adapt them to the stress caused during injection. Compared to the vehicle injection, body weight as well as food intake in WT and *BBS4^tg^* mice are decreased upon leptin injection. Although body weight decreased in *BBS4^tg^* and WT mice but not in *Bbs4^−/−^* mice upon leptin injection, the change was not significant due to low N, and higher variation in mouse weight. However, when compared to vehicle injection, a significant reduction in food intake in WT (*P<0.05) and *BBS4^tg^* (Ψ <0.05) mice were observed after leptin injection.
(TIF)

Figure S3 **Baseline renal sympathetic nerve activity (RSNA), hydrocephalus and motile cilia are improved in transgenic mice.** **A)** Three mice (9–12 weeks) per genotype group were used to measure the baseline renal RSNA. Baseline RSNA is increased in the *Bbs4^−/−^* mice compared to the WT mice. *BBS4^tg^* mice have normal renal RSNA. **B)** SEM image of lateral ventricles in WT, *Bbs4^−/−^*, and *BBS4^tg^* mice. Compared to WT and *BBS4^tg^*, *Bbs4^−/−^*mouse brain has very few motile cilia in the lateral ventricle. C) MRI image showing enlarged LV (red arrowhead) in the sagittal section of *Bbs4^−/−^* compared to WT and *BBS4^tg^* mouse brain.
(TIF)
