# Supplementary figures and images for: Ectopic Expression of Human BBS4 Can Rescue Bardet-Biedl Syndrome Phenotypes in Bbs4 Null Mice
Source: PLoS One. 2013 Mar 15;8(3):e59101. doi: 10.1371/journal.pone.0059101 (PMC3598656; doi:10.1371/journal.pone.0059101)

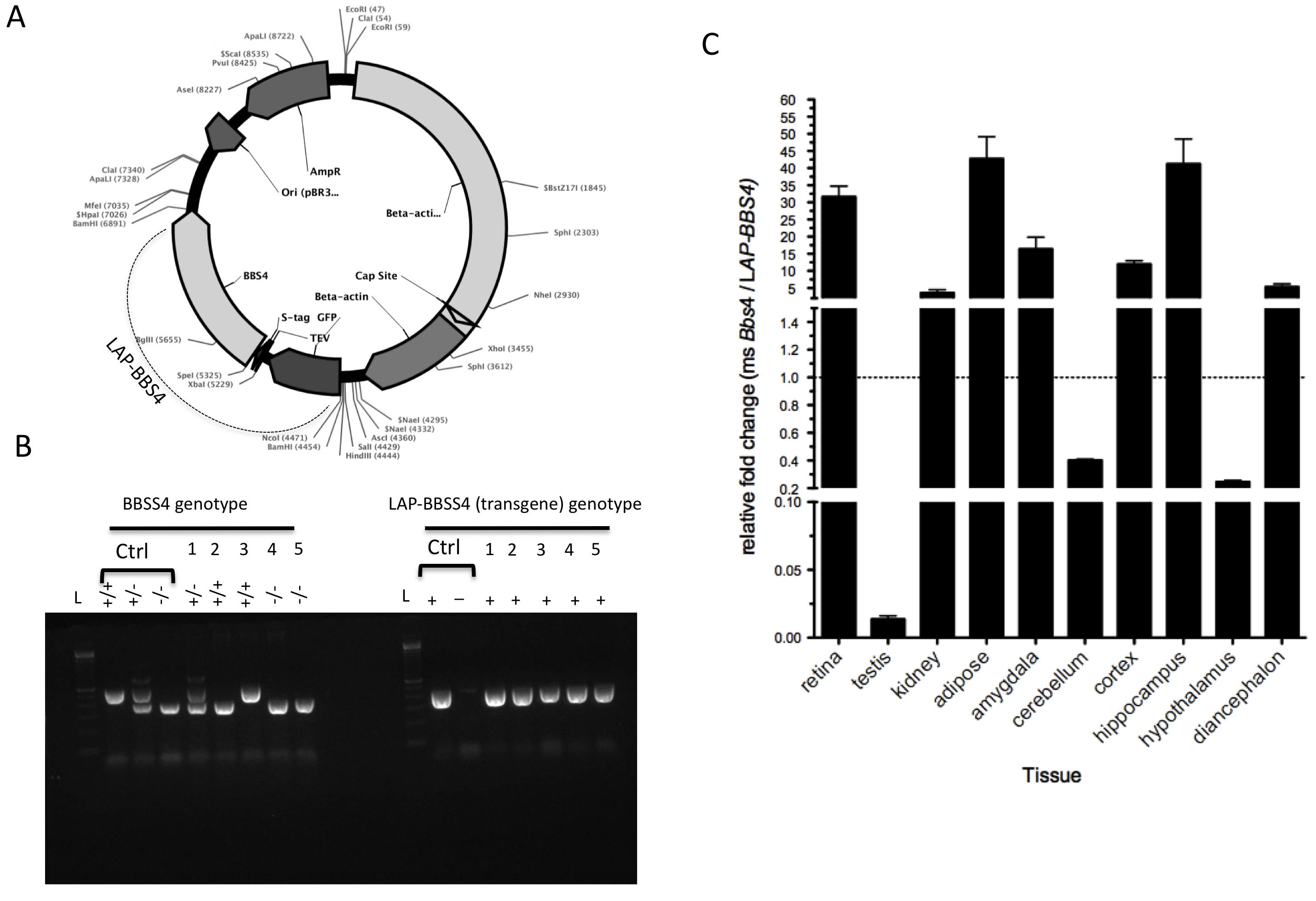

Supplement: Figure S1 — Genotypes and relative expression of transgene varies among tissues. A) Expression cassette of LAP-BBS4, where a LAP-tag is at the N-terminus of the BBS4 gene. LAP-BBS4 was cloned in phßAPr expression vector. B) Example of genotyping performed on the animals. Each animal was tested for the presence of the transgene as well as the endogenous Bbs4 gene. Left side represents genotype for endogenous Bbs4, and the right side is the genotype for the transgene. C) Graph showing relative expression of the transgene (LAP-BBS4) compared to the endogenous Bbs4. The Y-axis is the relative fold change (LAP-BBS4/msBbs4), and the X-axis shows tissues that were used. Horizontal dotted line at 1 in Y-axis represents the point where equal expression of the transgene and endogenous Bbs4 are observed. Above the line represents higher expression of LAP-BBS4 compared to endogenous Bbs4, and below that reference line shows higher expression of endogenous Bbs4 than transgene. For example, retina has relatively higher expression of endogenous Bbs4 than LAP-BBS4, and higher amount of LAP-BBS4 than the endogenous Bbs4 is expressed in testis. (TIF) [file pone.0059101.s001.tif]

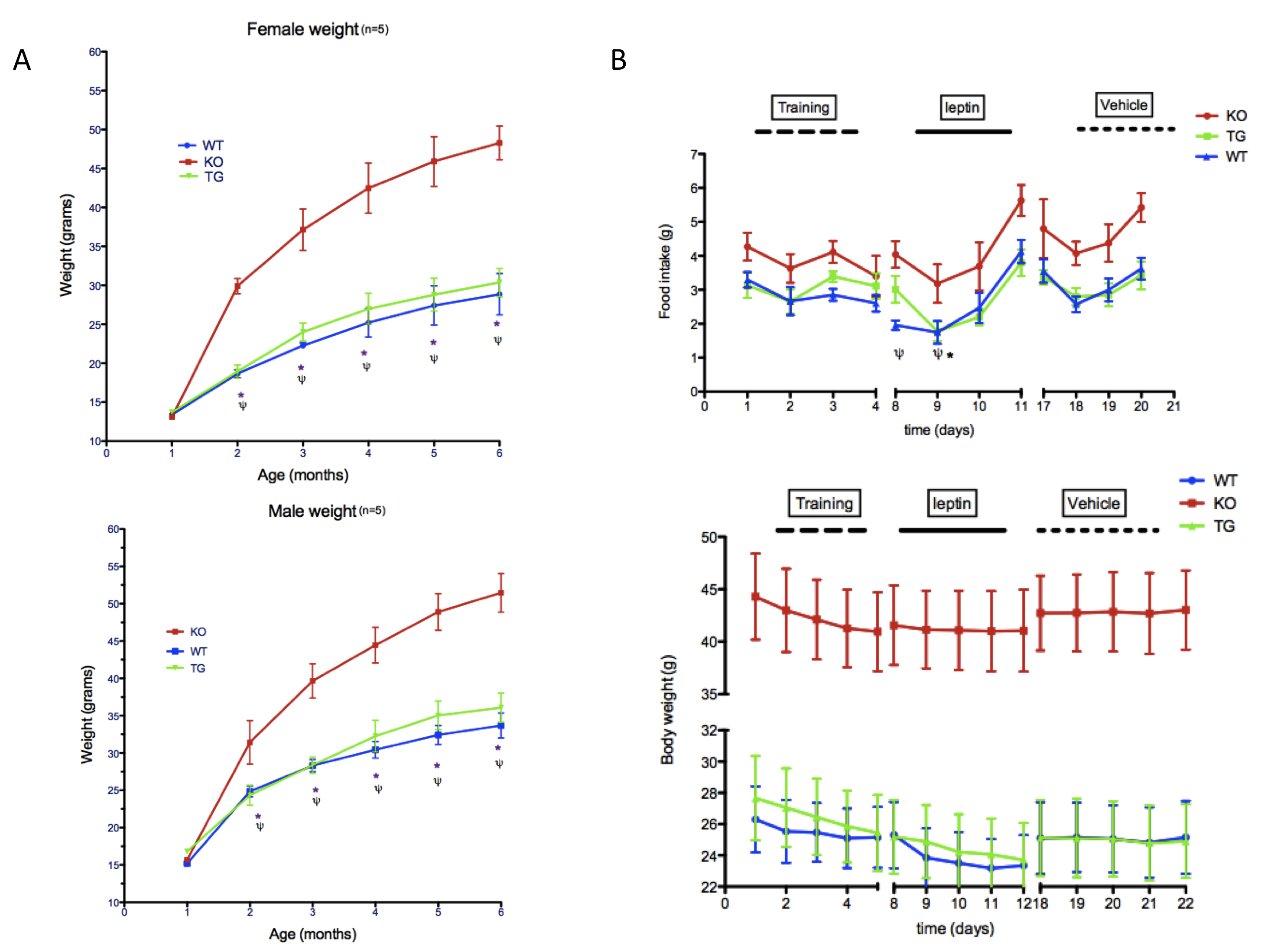

Supplement: Figure S2 — Obesity and leptin resistance in Bbs4tg mice compared to Bbs4−/− mice. A) 5 male and 5 female mice were weighed every month for 6 months. Bbs4−/− mice (red line) are significantly heavier than WT (*P<0.01)(green) and BBS4tg mice (Ψ P<0.01) beginning at 2 months of age. There is no significant difference in weight between Bbs4tg and WT mice in either male or female. B) Food intake and body weight were monitored in 4 mice (2 male 2 female each, 9–12 weeks old) of all three genotypes. Mice were kept in separate cages for a week followed by a training session of mock injection to adapt them to the stress caused during injection. Compared to the vehicle injection, body weight as well as food intake in WT and BBS4tg mice are decreased upon leptin injection. Although body weight decreased in BBS4tg and WT mice but not in Bbs4−/− mice upon leptin injection, the change was not significant due to low N, and higher variation in mouse weight. However, when compared to vehicle injection, a significant reduction in food intake in WT (*P<0.05) and BBS4tg (Ψ <0.05) mice were observed after leptin injection. (TIF) [file pone.0059101.s002.tif]

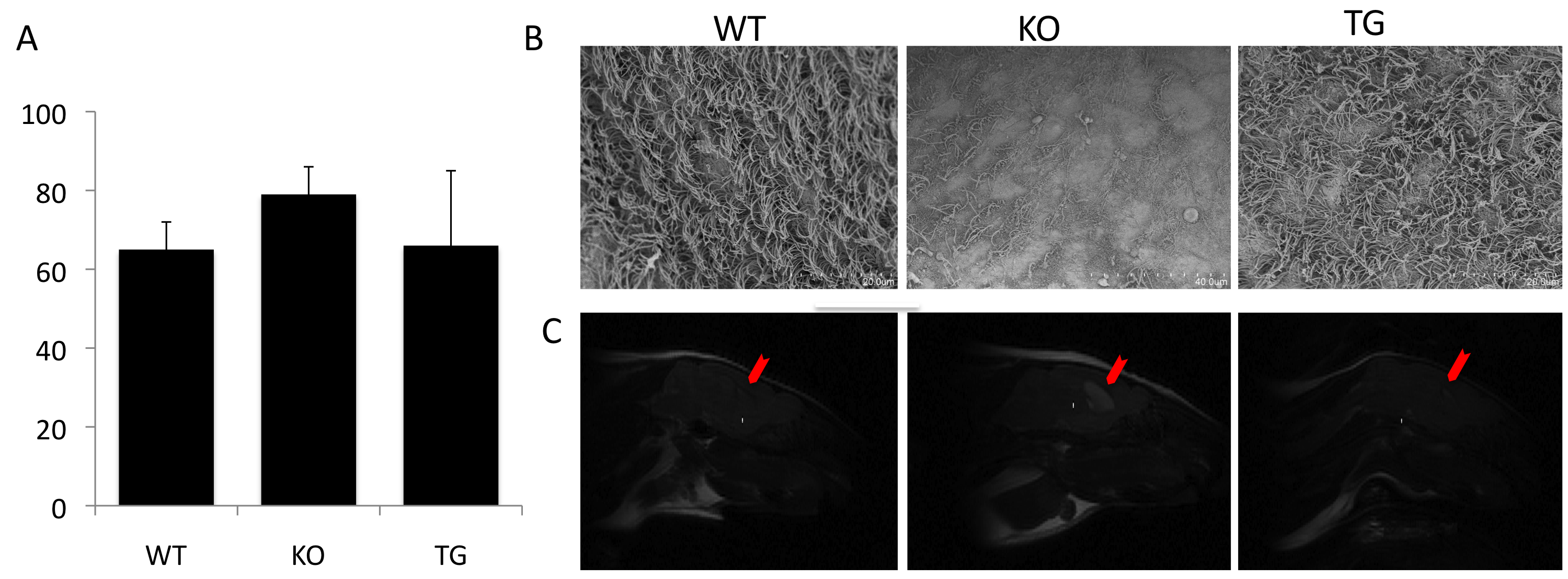

Supplement: Figure S3 — Baseline renal sympathetic nerve activity (RSNA), hydrocephalus and motile cilia are improved in transgenic mice. A) Three mice (9–12 weeks) per genotype group were used to measure the baseline renal RSNA. Baseline RSNA is increased in the Bbs4−/− mice compared to the WT mice. BBS4tg mice have normal renal RSNA. B) SEM image of lateral ventricles in WT, Bbs4−/−, and BBS4tg mice. Compared to WT and BBS4tg, Bbs4−/−mouse brain has very few motile cilia in the lateral ventricle. C) MRI image showing enlarged LV (red arrowhead) in the sagittal section of Bbs4−/− compared to WT and BBS4tg mouse brain. (TIF) [file pone.0059101.s003.tif]
